# Tactile Perception Technologies and Their Applications in Minimally Invasive Surgery: A Review

**DOI:** 10.3389/fphys.2020.611596

**Published:** 2020-12-23

**Authors:** Chao Huang, Qizhuo Wang, Mingfu Zhao, Chunyan Chen, Sinuo Pan, Minjie Yuan

**Affiliations:** ^1^ Institute of Computing Technology, Chinese Academy of Sciences, Beijing, China; ^2^ Ningbo Institute of Information Technology Application, Chinese Academy of Sciences, Ningbo, China

**Keywords:** tactile sensors, tactile perception, tactile images, minimally invasive surgery, robotic surgery, artificial intelligence

## Abstract

Minimally invasive surgery (MIS) has been the preferred surgery approach owing to its advantages over conventional open surgery. As a major limitation, the lack of tactile perception impairs the ability of surgeons in tissue distinction and maneuvers. Many studies have been reported on industrial robots to perceive various tactile information. However, only force data are widely used to restore part of the surgeon’s sense of touch in MIS. In recent years, inspired by image classification technologies in computer vision, tactile data are represented as images, where a tactile element is treated as an image pixel. Processing raw data or features extracted from tactile images with artificial intelligence (AI) methods, including clustering, support vector machine (SVM), and deep learning, has been proven as effective methods in industrial robotic tactile perception tasks. This holds great promise for utilizing more tactile information in MIS. This review aims to provide potential tactile perception methods for MIS by reviewing literatures on tactile sensing in MIS and literatures on industrial robotic tactile perception technologies, especially AI methods on tactile images.

## Introduction

Minimally invasive surgery (MIS) is a surgery approach that provides indirect access to anatomy for surgeons by introducing specially designed surgical instruments or flexible catheters into a patient’s body through minimally sized incisions (Verdura et al., 2000). Compared to conventional open surgery, MIS offers many advantages including reduced anesthesia and hospitalization time, mitigated tissue trauma and risk of postoperative infection, decreased intraoperative blood loss, and accelerated recovery (Puangmali et al., 2008). However, the indirect access to the anatomy brings two challenges: low degree of freedom (DOF) during manipulation and absence of tactile feedback during tool–tissue interactions (Abushagur et al., 2014). With the development of mechatronics, robot-assisted minimally invasive surgery (RMIS) systems, such as the ZEUS Surgical System (Uranues et al., 2002) and the da Vinci Surgical System (Guthar and Salisbury, 2000), have been developed to improve the dexterity of tools during manipulation, which partly resolve the motion constrain problem. Despite this, there are still limitations existing for MIS, including reduced hand-eye coordination, a narrowed field of vision, and limited workspace of the tools (Bandari et al., 2020). More importantly, surgeons have little tactile information in MIS compared to the rich tactile feedback of human hands, which severely impairs the surgeon’s ability to control the applied forces, thus causing extra tissue trauma or unintentional damage to healthy tissue (Ahmadi et al., 2012).

Tactile feelings, including but not limited to force, distributed pressure, temperature, vibrations, and texture, are complicated information that a human obtains through cutaneous receptors during physical interaction with environment. Depending on the sensing modalities, tactile sensors can be categorized into different kinds, including force sensors for measuring contact forces, slippage sensors for detecting slippage between tissue, and surgical instruments vibration sensors for measuring vibrations during contact. The goal of tactile technologies in MIS is to restore all the tactile information so that surgeons feel they are contacting that patients’ anatomy directly with their own hands rather than operating a mechanism. Among this tactile information, force data are relatively easy to acquire, model, quantify, and display, so it is most widely used in MIS. The sensing principles, design requirements, specifications, developments of force sensors, and their applications in MIS have been thoroughly reviewed (Eltaib and Hewit, 2003; Puangmali et al., 2008; Schostek et al., 2009; Tiwana et al., 2012; Abushagur et al., 2014; Konstantinova et al., 2014; Saccomandi et al., 2014; Park et al., 2018; Al-Handarish et al., 2020; Bandari et al., 2020). In contrast, studies on utilizing other tactile information in MIS are very rare. Researchers have begun to realize the advantages of various tactile information in MIS, but challenges remain. Van der Putten et al. found that slippage and texture information can augment force information to prevent tissue trauma during manipulation but limited by cost and changes in instability; few studies were about texture information (Westebring-van der Putten et al., 2008). Okamura found some studies on tactile sensor arrays to perceive pressure distribution or deformation over a contact area, but it was challenging to acquire and display tactile data due to size and weight constraints (Okamura, 2009).

Tactile sensors are often categorized into single-point tactile sensor and the tactile array with respect to their spatial resolution. The single-point tactile sensor is usually embedded in the tip of the equipment to confirm the object-sensor contact and detect tactile signals at the contact point. The tactile array is composed of several single-point tactile sensors arranged according to certain rules. Compared with single-point tactile sensors, tactile array sensor can cover a wider area and can capture the tactile information of the object from multiple dimensions, so it can achieve high spatial resolution of touch.

In the field of industrial robots, tactile perception technologies have received considerable attention. Tactile perception is a procedure that obtains tactile information from tactile data sensed by tactile sensors. Many methods have been proposed to accomplish robot tactile perception tasks, including shape recognition, texture recognition, stiffness recognition, and sliding detection (Liu et al., 2017). In the early years, single-point tactile sensors were used to create point cloud models to finish tactile perception tasks. A current trend of tactile perception researches is to represent tactile data as images, where a tactile element is treated as an image pixel. From tactile images that tactile sensor arrays acquired, features are extracted, such as statistical features, vision feature descriptors, principal component analysis (PCA)-based features, and self-organizing features (Luo et al., 2017). These features are usually processed by AI methods like clustering, support vector machine (SVM), and deep neural networks, to obtain tactile information.

Robotically assisted surgery is a type of surgical procedure that is done using robotic systems. It was developed to overcome the limitations of pre-existing minimally invasive surgery and to enhance the capabilities of surgeons performing open surgery. According to their level of autonomy, surgical robotic systems are often classified into two categories: autonomous systems, which automatically execute tasks without interventions of the practitioner, and nonautonomous systems, which reproduce the surgeon’s motion in either a master/slave teleoperated configuration or a hands-on configuration (Okamura, 2009). Due to the technical complications and high demanded reliability, most surgical robots belong to the second category. However, the development of robot tactile perception is promising for autonomous robotic systems. In the last decades, sensors have become smaller, cheaper, and more robust. Enormous studies on industrial robots aimed to perceive tactile in small areas like fingertips, on which sensors are tiny. Some studies accomplish tactile perception tasks with sensors made of soft material. In MIS, tactile information is usually displayed in the form of raw tactile data, which demands extra analysis. These tactile perception studies make it possible to provide more intuitive tactile information (e.g., stiffness distribution map) for surgeons utilizing nonautonomous surgical robotic systems and offer potential designs of autonomous surgical robotic systems.

In this paper, we review literatures on tactile perception technologies in industrial robots and MIS in the last decades to analyze the advantages and feasibility of applying tactile perception methods on MIS, especially the state-of-the-art AI methods on tactile images. Similarly, the features and advantages of tactile sensors varying in sensing modalities are analyzed, together with their applications in MIS.

The remainder of this paper is organized as follows: *Tactile Sensors and Their Applications in MIS* introduced tactile sensors and their applications in MIS. In *Tactile Perception Algorithms in MIS*, tactile perception algorithms in MIS are reviewed. In *Tactile Perception Applications in MIS*, the feasibility of applying tactile perception methods on MIS is analyzed. In *Conclusion*, a summary of the challenges and perspectives hoped for the future with tactile perception in MIS is presented.

## Tactile Sensors and Their Applications in MIS

Tactile sensors are used to collect tactile data at the contact point between the surgical equipment and tissues. Depending on modalities of tactile signal, various physical properties (e.g., softness and roughness) of a tissue can be extracted from tactile data. Tactile feedback is then provided for surgeons based on these detected physical properties. In most of the literatures, force feedback is the main form of tactile feedback, and force sensors are the most widely used tactile sensors. Tactile sensors can be categorized into the single-point tactile sensor and the tactile array sensor. In this section, studies on providing force feedback with the above two kinds of tactile sensors are reviewed. Except for force sensors and force feedback, some novel tactile sensors and tactile feedback methods are investigated.

### Single-Point Tactile Sensor and Force Feedback

A single-point tactile sensor is usually embedded on the tip of the surgical equipment to confirm the object–sensor contact and detect tactile signals at the contact point. In MIS, force feedback is extremely important to doctors in the consideration of the various consistency of the tissue. The force feedback implies the active force applied to the operators’ hands directly where the active force is usually related to the reactive force from the tissue to the tools. Many studies investigated the different application scenarios of force feedback in MIS. We summarized related cases into knotting, insertion, and incision, which will be described in the later paragraphs. After that, we will expound on the importance of force feedback in the abovementioned cases by a series of relevant studies, while the comparison with visual force feedback will also be referred to. We also investigated the development of force feedback in a famous minimally invasive surgical robotic system named da Vinci robot. Finally, the limitation of force feedback in minimally invasive surgery has been given out.

#### Knotting

In the knotting situation through the laparoscopic procedures, the force feedback indicating the tension of the thread from the tip of the tools is extremely important to guarantee the firmness of the knots but prevent damage to the tissue. To sense the point force feedback from tool tips, load cells are commonly used, as the case in (Song et al., 2009). Moreover, in (Song et al., 2011), a load cell with fiber Bragg grating (FBG) sensors was applied to measure the tension of the thread, where FGB sensors are optical fiber sensors improving the accuracy by encoding the wavelength. Richards et al. utilized the force/torque at the grasper-side to calculate the grasping force (Richards et al., 2000). Fazal and Karsiti decomposed the reactive force happened during the insertion process into three types by a piezoelectric type one-dimensional sensor and mathematical statistics, which were the force generated due to the stiffness of the tissue, the friction force, and the cutting force, thus enabling us to analyze each type of force separately (Fazal and Karsiti, 2009). Mohareri et al. creatively passed the reactive force produced by one hand to another hand and improved the knotting accuracy to 98% (Mohareri et al., 2014).

#### Incision

Apart from the suture scenario, the incision situation is another indispensable part of minimally invasive surgery. Callaghan and McGrath designed a force-feedback scissor with button load cells attached to the scissor blades to measure the interforce between the blades and the tissue (Callaghan and McGrath, 2007). However, a load cell normally could only sense force from one axial and two moments; therefore, the more complicated design is considered in the later researches. In (Valdastri et al., 2006), an integrated triaxial force sensor was developed and attached to the cutting tool for fetal surgery. A similar design of 3-DOF sensors could be found in (Berkelman et al., 2003). In (Kim et al., 2015), a type of surgical instrument with force sensors of 4 DOFs was developed, which could be applied to measure the normal force and the tangential force from the tip of the tools by capacitive transduction principle.

#### Palpation

Another conspicuous application scenario of force feedback in minimally invasive surgery is palpation. Since the tumor is always stiffer than the surrounding skin, the pressure intensity on the tumor tends to be obviously larger; therefore, sensors with a single point of contact can detect tumors by palpation (Talasaz and Patel, 2013). Similarly, to detect abnormal masses in the breast, a tactile sensing instrument (TSI) was designed in (Hosseini et al., 2010) and applied in a simulated scenario with a certain detecting route, which was the transverse scan mode. By combining the stress variation curves of each line, users could determine the x‐ and y-axis coordinates of the abnormal masses. The stress variations of the sensor in the two cases that were operating manually and by a robot showed a similar pattern. Besides, a new tactile sensory system was developed in (Afshari et al., 2011) by combining the displacement sensor and the force sensor to determine the existence and detect the location of kidney stones during laparoscopy. Since the surface stiffness was proportional to the result of the force sensor as well as the displacement sensor, the stiffness could be presented by these two values and depicted by a curving line through the path on the surface of model. Besides, Yip et al. first developed a miniature uniaxial force sensor to do endocardial measurements (Yip et al., 2010). In the research of (Munawar and Fischer, 2016), an elastic spherical proxy regions was designed to sense the forces from various directions.

#### Necessity of Force Feedback

To support the multiple cases in the above paragraphs, we also investigated the necessity of force feedback in minimally invasive surgery, which was proved and explained in (Morimoto et al., 1997) and (Tholey et al., 2005). The accuracy of the force applied seemed to be improved with the increasing force feedback in (Wagner and Howe, 2007) and (Bell et al., 2007). The reasons could be generalized into two points as in (Mohareri et al., 2014). One is sensing the invisible property such as the stiffness and the texture. Another one is preventing the undesired damage of tissue. Many studies investigated the cases of providing force feedback, visual feedback, visual force feedback (force feedback in the forms of image, sometimes like color bars), and no feedback. Mahvash et al. put forward the result that providing force feedback generated less error than other cases in the cardiac palpation (Mahvash et al., 2008). A similar result could be found in (Kitagawa et al., 2005). Mohareri et al. found out that the tightening degree in the knitting situation tended to be less uniform with visual feedback and summed up that the visual feedback could compensate part of the force feedback but was entirely not enough while applying the needles and thread (Mohareri et al., 2014). Reiley et al. investigated the practicability of the visual force feedback and concluded that operators without robotic experience could benefit from visual force feedback while practitioners do not as much as their counterparts (Reiley et al., 2008). Similar results were also shown in (Gwilliam et al., 2009) and summarized in (Okamura, 2009). However, visual force feedback could be the better solution in knot-tightening tasks as demonstrated in (Talasaz et al., 2012) and (Talasaz et al., 2017). Later, after (Talasaz et al., 2012), Talasaz and Patel first operated the system with an MIS tactile sensing probe remotely and viewed the feedback through a camera display (Talasaz and Patel, 2013). Besides, Guo et al. applied visual force feedback in vascular interventional surgery and showed great conformity (Guo et al., 2012).

Based on the aforementioned techniques, many operation platforms for minimally invasive surgery have been developed, including Robodoc, Probot, Zeus, and the most recent one named da Vinci (Marohn and Hanly, 2004; Puangmali et al., 2008; Munawar and Fischer, 2016). The da Vinci operation system solved several major limitations in recent minimally invasive surgeries, including the need for hand motion feedback, hand–eye coordination, feeling hands inside the body, expanding the DOF, elimination of surgeon tremor, and variable motion scaling (Guthar and Salisbury, 2000). Many pieces of research were applying based on the da Vinci operation system; however, the force feedback has been added to this system only recently. The examples could be found in (Mahvash et al., 2008) and (Reiley et al., 2008).

#### Challenges

Force feedback has been very promising for a long while; however, it has also faced some unsolved problems. Although the force feedback provides better tumor localization performance and more precise suture and incision operation with straightforward quantitative measures, it can be somehow time consuming since the measurement from one point to another is low effective in the algorithm level as shown in (Talasaz and Patel, 2013). Apart from this problem, another problem from the force feedback method is the attenuation of the force signal since the surgical tools are always long and stiff. To solve this problem, force amplification is considered as shown in (Song et al., 2009). However, due to the unpredictable disturbance of the tissues, the small disturbing force might also be amplified leading to fatal maloperation. To solve this problem, several actions in the laparoscopic cholecystectomy procedure are described and modeled in spatial coordinates in (Pitakwatchara et al., 2006) to amplify the operation reactive force but remain the disturbing force. Nevertheless, the research remained on the theoretical level without any real model.

### Tactile Array Sensor

**Figure 1:**
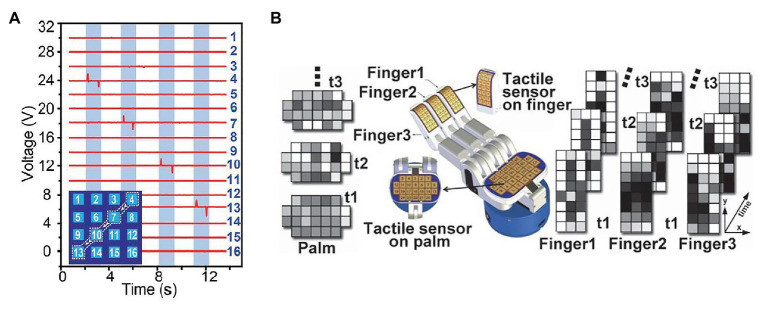
**(A)** An example of tactile wave diagram, where each waveform indicates voltage sensed by a sensing unit. This diagram shows the sensing result of a case that a capacitive stylus touched the surface of a 4 × 4 tactile array sensor along a path: 4 → 7 → 10 → 13 (Wang et al., 2016). **(B)** An example of tactile image sequences, where each sequence represent tactile data over time sensed by an tactile array sensor, and each image pixel represents tactile data sensed by a sensing unit in a certain time (Cao et al., 2016).

A tactile array sensor is composed of several single-point tactile sensors arranged according to certain rules. It is usually a flat cuboid with M × N tactile sensing units, where M and N indicate the number of rows and columns of sensing units. In the last decades, tactile data sensed by tactile array sensor was generally displayed as a wave diagram with M × N waveforms, each of which indicates a time-dependent physical quantity obtained by a sensing unit. Recently, with the development of computer version, the methods for processing images have been faster and more accurate, inspired by which tactile data are represented as image sequences, where each sequence represent tactile data over time sensed by a tactile array sensor, and each image pixel represents tactile data sensed by a sensing unit in a certain time. Figure 1 shows a comparison between a wave diagram and tactile images.

**Figure 2:**
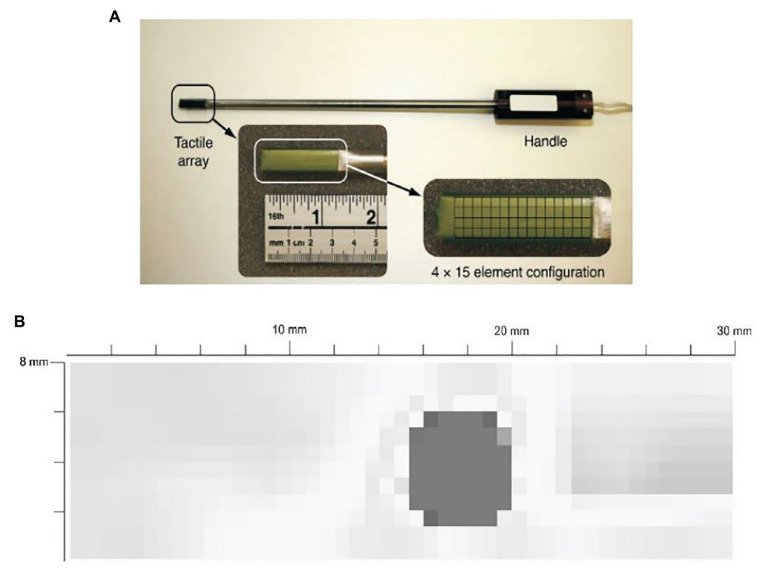
**(A)** A tactile sensor array with 4 × 15 sensing elements. **(B)** a typical contour map of a tumor obtained from the visualization software (Trejos et al., 2009).

For example, Trejos et al. developed a TSI that uses a commercially available pressure pad (Trejos et al., 2009). The TSI is shown in Figure 2A. The TSI industrial TactArray on this instrument consists of an array with 15 rows and 4 columns of electrodes, which are oriented orthogonally to each other. Each overlapping area created by the row and column electrodes forms a distinct capacitor. The results from the tactile sensor as shown in Figure 2B converts the measured voltage values from the capacitive sensor to pressure measurements and displays these results in a color contour map of pressure distributions.

**Figure 3:**
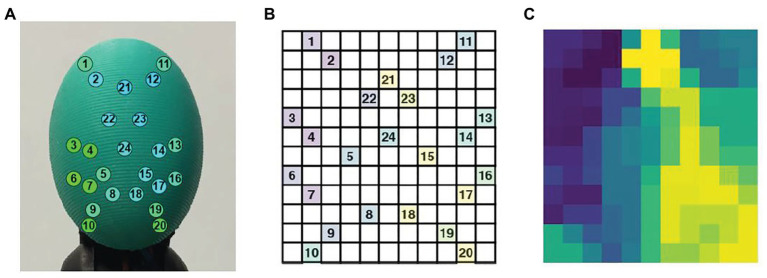
**(A)** The BioTac sensor with 24 electrodes distributions, **(B)** distribution of the BioTac SP electrodes in a 12 × 11 tactile image, **(C)** result of filling the gaps in the tactile image with the mean value of the eight-closest neighbors (Zapata-Impata et al., 2019).

Zapata-Impata et al. used the BioTac SP tactile sensor manufactured by Syntouch (Zapata-Impata et al., 2019). Figure 3A shows a representation of the location of the electrodes in the sensor. A tactile image can be created for this 2D array in which the 24 electrodes values $e_{i}$ are spatially distributed to occupy the image pixels at certain coordinates $\left(i,j\right)$. Basically, the tactile image consists of a 12 × 11 matrix in which the 24 electrodes are distributed as shown in Figure 3B. Figure 3C shows the final tactile image; all the gaps (cells without assigned values) are then filled using the mean value of the eight-closest neighbors.

**Figure 4:**
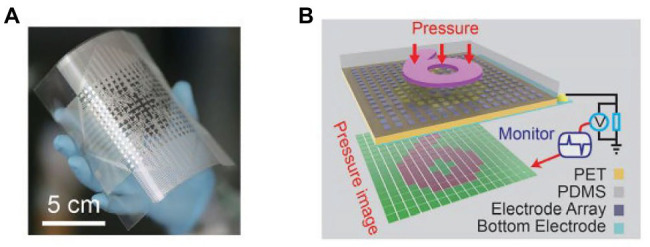
**(A)** Photograph of a fabricated 16 × 16 TESM with good flexibility. **(B)** Demonstration of the mapping output voltage of the sensor matrix under the pressure of a module in the shape of a “6” (Wang et al., 2016).

Wang et al. reported a self-powered, high-resolution, and pressure-sensitive triboelectric sensor matrix (TESM) based on single-electrode triboelectric generators that enable real-time tactile mapping (Wang et al., 2016). Figure 4A shows a flexible 16 × 16 pixelated TESM with a resolution of 5 dpi can map single and multipoint tactile stimuli in real time *via* the multichannel data acquisition method while maintaining an excellent pressure sensitivity of 0.06 kPa^−1^ and long-term durability. Figure 4B is a schematic of how the sensor matrix images the pressure distribution when a mold in the shape of a “6” is pressed against the top of the TESM.

Compared with single-point tactile sensors, a tactile array sensor can cover a wider area and can capture the tactile information of the target from multiple dimensions, so it can achieve high spatial resolution of touch. Therefore, it is applied to minimally invasive surgery now. For vascular interventional surgery, Guo et al. reported a novel catheter sidewall force tactile sensor array, which is based on a developed robotic catheter operating system with a master–slave structure (Guo et al., 2013). It can detect the force information between the sidewall of the catheter and the blood vessel in detail and transmit the detected force information to the surgeon through the robot catheter system. Besides, to reduce the postoperative pains, Li et al. proposed an original miniature three-dimensional force sensor that can detect the interaction forces during tissue palpation in minimally invasive surgery (Li et al., 2015). In addition, to detect and locate tissue abnormalities, Li et al. presented a novel and high-sensitivity optical tactile sensor array based on fiber Bragg grating (FBG) (Li et al., 2018). Each tactile unit is mainly composed of a spiral elastomer, a suspended optical fiber engraved with an FBG element, and a contact connected with elastomers with threads. Moreover, for tissue palpation, Xie et al. proposed a new type of optical fiber tactile probe, which consists of 3 × 4 tactile sensors (Xie et al., 2013). In this paper, one single camera is employed to capture and detect the light intensity changes of all sensing elements and convert to force information. Finally, for tissue palpation, Roesthuis et al. proposed an experimental bench, which includes a tendon-driven manipulator. A kind of nitinol FBG wire is fabricated, on which 12 FBG sensor arrays are integrated and distributed over four different groups. In closed-loop control, the minimum average tracking error of circular trajectory is 0.67 mm (Roesthuis et al., 2013).

### Novel Tactile Sensor and Tactile Feedback

#### Novel Tactile Sensor

In conventional open surgery, surgeons make ample use of their cutaneous senses to differentiate tissue qualities, which can hardly be achieved with force sensors alone, motivating some researchers to expend effort on enabling other sensing modality in MIS. In the last decades, researchers have made an attempt to use other tactile signals to measure properties of tissues. Eklund et al. developed an *in vitro* tissue hardness measurement method using a catheter-type version of piezoelectric vibration sensors (Eklund et al., 1999). Eltaib and Hewit proposed a tactile sensor by attaching a pressure sensor to the end of a sinusoidally driven rod of the tactile probe. The sensor measured both vibration and contact force to detect differences between soft and hard tissues and assist surgeons in detecting abnormal tissues (Eltaib and Hewit, 2000). Baumann et al. presented a method of measuring mechanical tissue impedance by determining resonance frequency with an electromechanic vibrotactile sensor integrated into an operating instrument (Baumann et al., 2001). Chuang et al. reported a miniature piezoelectric hardness sensor mounting on an endoscope to detect submucosal tumors (Chuang et al., 2015). Kim et al. fabricated sensorized surgical forceps with five-degree-of-freedom (5-DOF) force/torque (FIT) sensing capability (Kim et al., 2018). A summary of the representative tactile sensors and their applications is presented in Table 1.

**Table 1:** Representative tactile sensors and their applications.

Literature	Application	Sensor type
Eklund et al., 1999	*In vitro* tissue hardness measurement	Catheter type version of piezoelectric vibration sensor
Baumann et al., 2001	Measuring mechanical tissue impedance	Electromechanic vibrotactile sensor
Song et al., 2009	Measuring the tension of the thread	Single-point FBG force sensor
Trejos et al., 2009	Assessing the feasibility of using the tactile sensing instrument under robotic control to locate underlying tumors.	Six-DOF force/torque sensor array
Hosseini et al., 2010	Detecting abnormal masses in the breast	Tactile probe
Afshari et al., 2011	Detecting the location of kidney stones	Force sensor and displacement sensor
Mohareri et al., 2014	Passing the reactive force produced by one hand to another hand for bimanual robot-assisted surgery	Single-point force sensor
Chuang et al., 2015	Detecting submucosal tumors	Piezoelectric hardness sensor
Kim et al., 2015	Measuring the normal force and the tangential force from the tip of the tools in the incision situation	Four-DOF force sensor
Wang et al., 2016	Real-time tactile mapping	Triboelectric sensor array
Li et al., 2018	Detecting and locating tissue abnormalities	Optical tactile sensor array

#### Tactile Feedback

Some novel tactile display systems were developed to provide feedback for surgeons based on various tactile information. Schostek et al. proposed a tactile sensor, integrated into a laparoscopic grasper jaw, to obtain information about shape and consistency of tissue structures (Schostek et al., 2006). The tactile data were wirelessly transferred *via* Bluetooth and graphically displayed to the surgeon. However, tissue exploration time was longer compared to a conventional grasper. Prasad et al. presented an audio display system to relay force information to the surgeon, but continual noise in an operating room setting remained a problem (Prasad et al., 2003). Fischer et al. developed a system that displayed oxygenation values to surgeons. They simultaneously used force sensors and oxygenation sensors to measure tissue interaction forces and tissue oxygenation next to translational forces, when tissue oxygenation decreases below a certain value, trauma will occur (Fischer et al., 2006). Pacchierotti et al. reported a cutaneous feedback solution on an da Vinci surgical robot. They proposed a model-free algorithm based on look-up tables to map the contact deformations, dc pressure, and vibrations to input commands for the cutaneous device’s motors. A custom cutaneous display was attached to the master controller to reproduce the tactile sensations by continually moving, tilting, and vibrating a flat plate at the operator’s fingertip (Pacchierotti et al., 2016).

## Tactile Perception Algorithms in MIS

Recent researches on tactile perception algorithms are focused on the tactile array sensor. With the tactile array sensor, we can collect an *M* × *N* tactile image, where each tactile element is treated as an image pixel. From tactile images that tactile sensor arrays acquired, features are extracted, such as statistical features, vision feature descriptors, PCA-based features, and self-organizing features. These features are usually processed by AI methods like clustering, SVM, and deep neural networks to obtain tactile information. After training the algorithm, we can use it to assist doctors in minimally invasive surgery. There are a lot of scenarios where the algorithm can be used. We summarized related cases into wall following, shape recognition, stable scraping, and hardness detection.

### Wall Following

To perform wall following, Fagogenis et al. designed an image classifier, which is based on machine learning and can distinguish between blood (no contact) or ventricular wall tissue and the bioprosthetic aortic valve (Fagogenis et al., 2019). The algorithm used the bag-of-words method to group tactile images, which is based on the number of occurrences of specific features of interest. During training, the algorithm can select features that were of interest and the relationship between their number and the tactile image class. For training, they used OpenCV to detect features in a set of training images based on manually labeled. Then, the detected features are mathematically encoded with LUCID descriptors to achieve efficient online computation. In order to reduce the number of features, they used clustering (k-mean) to identify the optimal feature representatives. The resulting cluster centers were the representative features used for the rest of the training and for runtime image classification. After determining the representative feature set, they traversed the training data for the second time and constructed the feature histogram for each image by calculating the number of times each representative feature appeared in the image. The last step was to train an SVM classifier, which learned the relationship between the feature histogram and the corresponding classes. Using the trained algorithm, we first detected the features and calculated the corresponding LUCID descriptors and then classified the images. Then, these features were matched to the nearest representative features, and the resulting feature histogram was constructed. Based on the histogram, the SVM classifier is used to predict the tissue-based contact state. They used a small group of training tactile images (~2,000 images) with training taking just a few minutes (~4 min) and achieved good results. Because image classification took 1 ms, our haptic vision system estimated contact state based on the camera’s frame rate (~45 frames/s). The accuracy of contact classification algorithm is 97% (tested on 7,000 images not used for training) with type I error (false positive) of 3.7% and type II (false negative) of 2.3%.

### Shape Recognition

**Figure 5:**
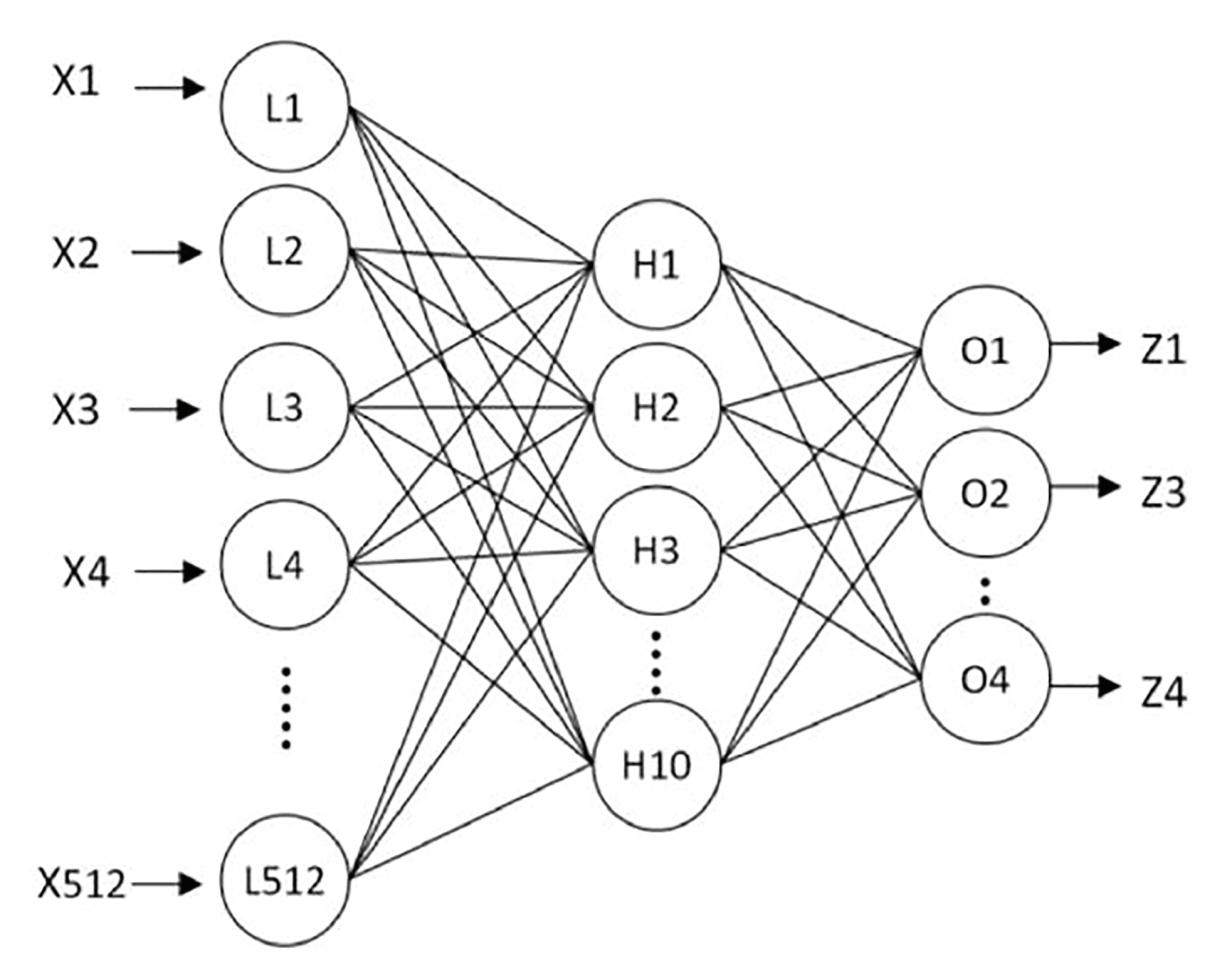
The three-layer neural network for object classification (Liu et al., 2012).

To recognize the shape of an object, Liu et al. proposed a new algorithm to identify the shape of an object by tactile pressure images, which can distinguish the contact shapes between a group of low-resolution pressure maps (Liu et al., 2012). The algorithm can be divided into four steps. The first step of the algorithm is “Data extraction.” Data extraction normalizes the strongly correlated tactile images sequence into a single map to save computational cost and reduce the disturbances from signal noise. The second step is “Preprocessing.” It consists of several subalgorithms to prepare the information for its latter “Feature extraction.” Preprocessing is essential not only to prepare the information for further steps but also to enhance the interests of tactile images. The third step is “Feature extraction.” In this step, the tactile image is transformed into a 512-feature vector, and the extracted features are used to train the developed neural network for object shape recognition. These features are not affected by occlusion, position, or scale, as well as image size, resolution, and number of frames. All these characteristics make the algorithm robust and effective. Finally, a three-layer neural network is developed to train, validate, and test the efficiency and success rate of the algorithm. It is trained to use the features extracted at the previous stage as a classifier. Figure 5 shows a diagram of the three-layer neural network for object classification. Through the experimental study, it was found that using four different contact shapes to test, the average classification accuracy reached 91.07%. The shape recognition algorithm based on the feature extraction has strong robustness and effectiveness in distinguishing different target shapes. It can be directly applied to minimally invasive surgery to identify the shape of the contact site and determine whether the tissue is abnormal, which is convenient for doctors to detect abnormal tissues with abnormal shapes in time.

### Stable Scraping

**Figure 6:**
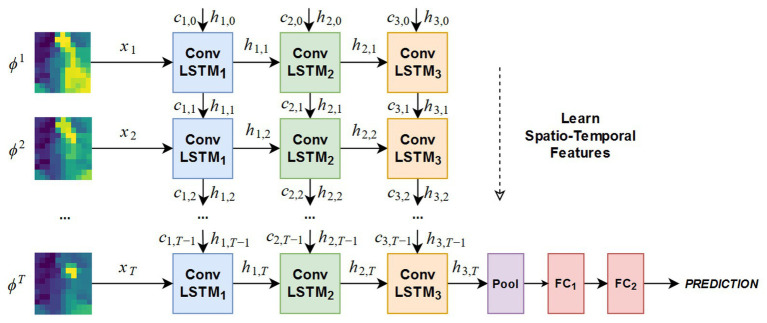
Architecture of the ConvLSTM network tested in the experimentation (Zapata-Impata et al., 2019).

To judge the stability of the grip, Zapata-Impata et al. proposed a spatiotemporal tactile features learning method based on convolutional long short-term memory (ConvLSTM) (Zapata-Impata et al., 2019). This method preprocessed the tactile readings and fed to a ConvLSTM that learns to detect multiple types of slip directions with just 50 ms of data. The architecture of the ConvLSTM network is shown in Figure 6. For preprocessing data, this method used a sensor with 24-electrode distributions to obtain tactile images. In more detail, the sensor uses these electrodes to record signals from four emitters and measure the impedance in the fluid between them and the elastic skin of the sensor. The fluid moves while contact is experienced by these sensors, thus affecting the measurements made by the electrodes. The whole method used four object sets, containing a total of 11 different objects, and was used to capture a new tactile dataset, recording seven different types of slip directions: north, slip south, slip east, slip west, slip clockwise, slip anticlockwise, or touch. Basically, the method created the ConvLSTM learns spatial features from pictures while simultaneously learning temporal ones. In the process of creating ConvLSTM, this method studied how the performance of the ConvLSTM changes depending on several parameters: the number of ConvLSTM layers, the size of the convolutional filters, and the number of filters inside each ConvLSTM layer. Finally, according to the experimental results, the network structures of five ConvLSTM layers, 3 × 3 filters, and ConvLSTM layers with 32 filters are selected to focus more attention on the low-level details in the tactile image and get better accuracy. For feature learning in time series, this method only needs three to five continuous tactile images, and the network can accurately learn to detect the sliding direction. In the task of detecting these seven states on seen objects, the system achieved an accuracy rate of 99%. Even if the ConvLSTM network was sensitive to new objects, during the robustness experiments, its performance dropped to an accuracy rate of 82.56% in the case of new objects with familiar properties (solids set) and an accuracy rate of 73.54 and 70.94% for stranger sets like the textures and small sets. The spatiotemporal tactile features learning method can be directly applied to minimally invasive surgery to improve the stability of tissue detect/mass grasp. However, at present, the single-point sensor is used to judge the grasping stability, and the judgment of the slip direction is only based on the single-point tactile characteristics. If the array tactile map is used, the regional feature information can be considered in the process of judgment to improve the stability of grasping. Therefore, it is very promising to apply this algorithm to minimally invasive surgery.

### Hardness Detection

**Figure 7:**
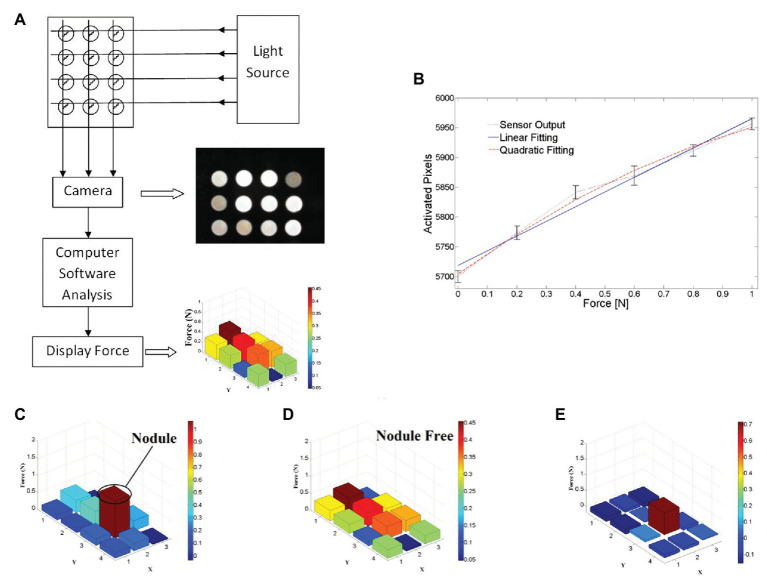
**(A)** Schematic design of proposed tactile sensor using camera, **(B)** measured output responses of sensing element 1 to the normal force applied, **(C)** test results on nodule area, **(D)** test results on nodule-free area, and **(E)** effective stiffness distribution map by compare test result on two areas (Xie et al., 2013).

To detect hardness, Yuan et al. designed a deep learning method that can estimate shape-independent hardness (Yuan et al., 2017). The algorithm, the convolutional neural network (CNN) feature layer of VGG network, is used to extract the physical signs of the tactile image, and a feature sequence is generated and input to LSTM to evaluate the softness and hardness of the sample. The algorithm can estimate the hardness of objects with different shapes and hardness ranging from 8 to 87 in Shore 00 scale. In minimally invasive surgery, tissue hardness detection is very important. Tumor detection is a very good example: some solid tumors are harder than the surrounding tissue, and their existence can also be obtained through tactile feedback to determine the location of resection and increase the success rate of surgery. Xie et al. proposed a method based on pixel calculation to measure the normal force and its distribution in the sensor area, to judge the hardness range of the area and determine the abnormal structure (Xie et al., 2013). As shown in Figure 7, in this method, the tactile image data of different brightness under different forces are captured by an optical fiber tactile sensor containing 3 × 4 sensing elements, and the tactile image is divided into 12 different regions in turn. By calculating the pixel values of each sensing region, and according to the predetermined linear relationship, the magnitude of the force applied in the region is obtained. The sensor outputs responses after palpation in two different areas; Figure 7C shows areas including the nodule, while Figure 7D shows areas that do not. From the result, outputs of each sensing element in the nodule-free area vary mostly in the range of 0–0.4 N. while in the nodule-embedded area, outputs of the sensing elements in contact with the nodule exceed the value of 0.8 N. The location of the nodules can be seen more clearly by subtracting Figure 7C of Figure 7D from Figure 7E. This method determines the tissue lump according to the pressure distribution map; although it is effective in some ways, the method based on linear fitting has a risk of producing large errors, and the definition range of hardness is single. If the image processing algorithm is used, the above two defects can be improved. Therefore, in the tissue hardness detection method, the method based on array tactile image processing is worth studying.

## Tactile Perception Applications in MIS

In the minimally invasive surgery, given the very small holes for the tools to operate, the capacity to feel tends to be limited as discussed in (Eltaib and Hewit, 2003). In line with that, Dargahi and Najarian summarized four categories of properties that were usually considered to be important in minimally invasive surgery including the force, position and size, hardness/softness, and roughness and texture (Dargahi and Najarian, 2004). However, with the help of tactile perception, the general performance is liable to be improved. To show the feasibility of the assistance provided by the tactile perception in the minimally invasive surgery, we will discuss the assistance provided by tactile perception catering for each property, respectively, after which the general discussion will be given.

### Obtaining Tactile Properties of Tissues

Measuring the acting and reactive force from tissues could be applied in many cases such as controlling the surgical knife on the liver tissue (Chanthasopeephan et al., 2003), measuring the tension of the thread while knotting (Song et al., 2009), modeling needle insertion force (Fazal and Karsiti, 2009), and differentiating between tissue samples in the scissoring process (Callaghan and McGrath, 2007). The doctors usually rely on the magnitude of force feedback to estimate when to stop every single shearing or insertion operation. For example, He et al. designed a 3-DOF force sensing pick instrument applied in the retinal microsurgery with fiber optic sensors placed at the distal tip of the surgical instrument. To realize multiple degrees of freedom, a linear model and second-order Bernstein polynomial were used to distinct forces in different directions (He et al., 2014). Besides, many previous studies like (Mohareri et al., 2014) show that, with the force-feedback data, doctors tend to make each separate operation more uniform, such as knots with similar thread tension. Nevertheless, the sensing directions and the accuracy are still of the top concern when researchers strive to improve the overall performance of the assistance of force.

**Figure 8:**
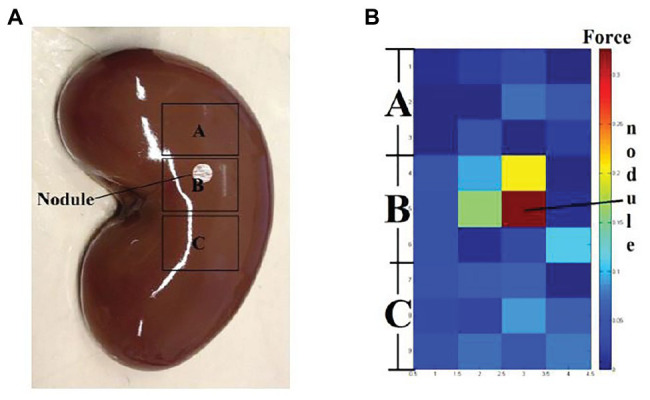
**(A)** A kidney with invisible nodule buried in area B. **(B)** The sensing result by palpating on three areas. Various color blocks indicating different force values (Xie et al., 2013).

The assistance in position and size is usually for tumor localization (Hosseini et al., 2006; Perri et al., 2010). For example, Liu et al. measured the indentation depth to detect the abnormal part of the tissue (Liu et al., 2010). Lederman et al. used rigid fingertip sheaths to locate the 3D mass (Lederman and Klatzky, 1999). Afshari et al. utilized the stress distribution to determine the stone inside the kidney (Afshari et al., 2010). Most of the time, the position and size of mass are reflected by the surging magnitude of rigidity sensed from the tools during palpation or the image constructed by an array of force sensors. In (Xie et al., 2013), a 3 × 4 sensing array was designed to detect the force distribution, and the doctors could be provided with the visualized data on which area tended to be stiffer. In the evaluation test on a lamb kidney with nodules embedded, the design presented a very effective performance. The doctors are more likely to rely on their experience to estimate the position and size based on the force feedback. Figure 8 shows an example of the mass localization utilizing an array of force sensors.

When calculating the hardness/softness, the sensors are usually placed on endoscopic graspers (Najarian et al., 2006). In many cases, tissue hardness could also be utilized to locate the mass in palpation. For instance, Ju et al. relied on the sensors on the catheter robot to locate the mass (Ju et al., 2018, 2019). Moreover, Kalantari et al. measured the relative hardness/softness of the tissue to sense various types of cardiac tissues while performing mitral valve annuloplasty (Kalantari et al., 2011). In (Yip et al., 2010), a creative uniaxial force sensor based on fiber optic transduction was developed, which could detect very small forces but show few root mean square (RMS) errors. In the designing process, properties of waterproof, electrical passivity, and material constraints were especially considered so that the instrument could perfectly meet the requirement of operating in the cardiac environment. However, there is no certain threshold to determine hard or soft by machine, which means the subjective judgment from doctors is indispensable.

Roughness and texture are the fourth groups of properties that can assist doctors in MIS. To measure roughness and texture, the sensors are usually placed on endoscopic graspers (Bonakdar and Narayanan, 2010), like laparoscopic graspers (Dargahi and Payandeh, 1998; Dargahi, 2002; Zemiti et al., 2006; Lee et al., 2016), which could be applied to cholecystectomy (Richards et al., 2000) and Nissen fundoplication (Rosen et al., 2001), and could also measure viscoelastic properties of tissues (Narayanan et al., 2006). In (Bicchi et al., 1996), tissue elastic properties were measured. For instance, in (Dargahi, 2002), a polyvinylidene fluoride tactile sensor was designed to measure the compliance and roughness of tissues. The principle was to measure the relative deformation when the tissue contacted with the sensor surface. However, it is never the best choice to detect all the tissue surface points by point, which indicates the large time consumed. For wiser utilization, subjective judgment by humans should also be taken into account.

### AI-Based Tactile Perception Applications in MIS

In line with the aforementioned cases, some researchers have devoted to the intelligent algorithms that can diminish the participation of subjectivity. Many of them have been described in *Tactile Perception Algorithms in MIS*. In this subsection, we will introduce more AI-based tactile perception technologies that were proven to be effective in MIS. Beasley and Howe used the pulsatile pressure variation from force sensors to find the artery through a signal processing algorithm and applied an adaptive extrapolation algorithm to generate the ultimate position prediction. The rough idea of adaptive extrapolation was applying 15 sensing samples and linear regression to fit the predicted arteries. It has been tested on the ZEUS Surgical Robot System and resulted in a < 2-nm mean error (Beasley and Howe, 2002).

**Figure 9:**
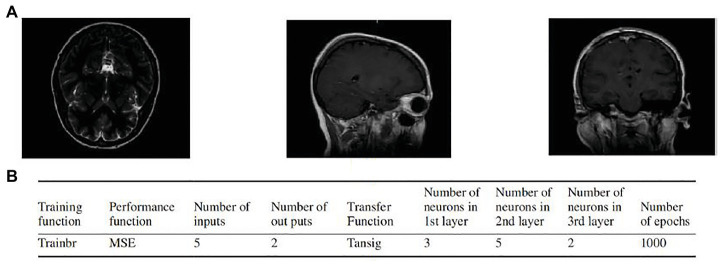
**(A)** Examples of the brain thermogram. **(B)** Parameters of artificial neural network (ANN; Sadeghi-Goughari et al., 2016).

Sadeghi-Goughari et al. introduced a new minimally invasive diagnosis technique named intraoperative thermal imaging (ITT) based on artificial tactile sensing (ATS) technology and artificial neural networks (ANNs) (Sadeghi-Goughari et al., 2016). In this study, a forward analysis and an inverse analysis based on ANN were proposed to estimate features including temperature and depth of a tumor using a brain thermogram. The brain thermogram is shown in Figure 9A. This work involved the forward analysis of heat conduction in cancerous brain tissue by employing a finite element method (FEM). Then, a three-layer feed-forward neural network (FFNN) with back propagation learning algorithm was developed to estimate related features of a tumor. Parameters of the proposed FFNN are shown in Figure 9B. The inputs of FFNN are thermal parameters extracted from tissue surface temperature profiles. Training of the ANN was performed by a backpropagation algorithm. By comparing estimated values of tumor features and expected values, potential brain tissue abnormalities were detected, which greatly facilitate the task of the neurosurgeon during MIS.

**Figure 10:**
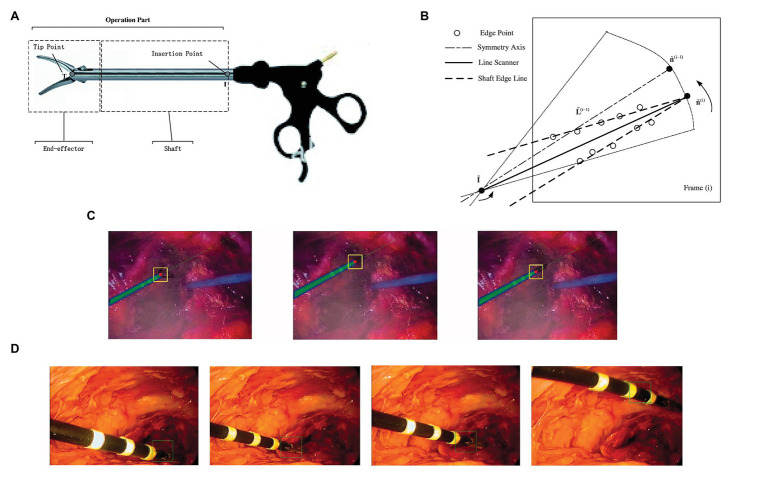
**(A)** The operation part of minimally invasive surgery (MIS) instrument: end effector and shaft portion; **(B)** line scanner application for detection of shaft edge lines and shaft image direction estimation; **(C)** selected frames of the instrument tracking and detection: the red circles are the tracked end-effector tip position, and the green dashed line is the shaft symmetry axis; **(D)** example frames of *in vivo* sequences with the end-effector positions shown by squares (Zhao et al., 2017).

Zhao et al. proposed a tracking-by-detection framework of surgical instruments in MIS (Zhao et al., 2017). As shown in Figure 10A, the operation of conventional MIS instruments can be subdivided into shaft portions and end effector. In the proposed method, the shaft portion was described by line features through the random sample consensus (RANSAC) scheme, and the end effector was depicted by some special image features based on deep learning through a well-trained CNN. With camera parameters and insertion points, a tracking method was proposed to estimate the 3D position and orientation of the instruments. As shown in Figure 10B, the scanning range was restricted to a sector area with the symmetry axis ${\tilde{L}}^{\left(i-1\right)}$, where $\tilde{I}$ is the image and s is an arbitrary scale. For the current frame i, the bounding box $\left(\tilde{p},s\right)$ slid along the symmetry axis ${\tilde{L}}^{\left(i\right)}$ obtained by shaft detection. The parameter $\tilde{p}$ is the center of the bounding box. The image in the bounding box at every sliding step with scale s was resized to 101 × 101 and then used as an input for the trained CNN. The highest score of the CNN positive output corresponds to the bounding box $\left({\tilde{p}}^{i},s^{i}\right)$, where ${\tilde{p}}^{i}$ is treated as the imaged tip position direction of the current frame i. Figure 10C shows the selected frames from the tracking procedure of the proposed method. However, compared with those in the *ex vivo* test, the 2D measurement error in the *in vivo* test was at least 2.5 pixels. When the respective 2D tracking by the proposed method was applied to each frame with the CNN-based detection of instruments, the insufficient illumination of the image part (end effector) accounted for drifted tracking results in some frames (see Figure 10D), which is the main reason why the *in vivo* test has higher 2D measurement errors. This issue can be resolved by adding samples of *in vivo* sequences into the training database.

**Figure 11:**
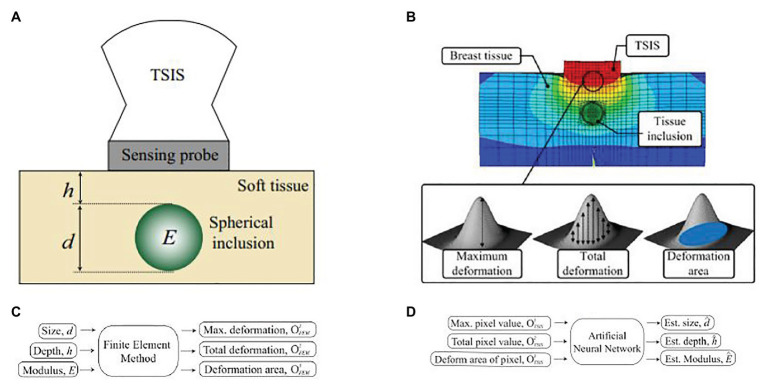
**(A)** A cross-section of an idealized breast model for estimating inclusion parameters. The tissue inclusion has three parameters: size *d*, depth *h*, and Young’s modulus *E*. **(B)** Finite element method (FEM) model of an idealized breast tissue model. The sensing probe of tactile sensation imaging system (TSIS) is modeled on top of the breast tissue model. **(C)** The forward algorithm. **(D)** The inversion algorithm (Lee and Won, 2013).

Lee and Won presented a novel method to estimate the stiffness and geometric information of a tissue inclusion (Lee and Won, 2013). The estimation was performed based on the tactile data obtained on the tissue surface. To obtain the tactile data, the author developed an optical tactile sensation imaging system (TSIS). The TSIS obtained tactile images with maximum pixel values, total pixel values, and deformation areas. These parameters were used to estimate the size, depth, and elasticity of the embedded lesions. The proposed method consisted of a forward algorithm and an inversion algorithm. The forward algorithm was designed to predict maximum deformation, total deformation, and deformation areas based on the parameters including size, depth, and modulus of the tissue inclusion. In the inversion algorithm, tactile parameters obtained from the TSIS and simulated values from the forward algorithm were used to estimate the size, depth, and modulus of the embedded lesion. Figure 11A describes a cross-section of an idealized breast mode. Figure 11B shows the sensing probe of TSIS modeled on top of the breast tissue. When the TSIS compressed against the tissue surface containing a stiff tissue inclusion, it produced different parameters: size d, depth h, and Young’s modulus E. The FEM in the forward algorithm quantified deformation as the maximum deformation $O_{\mathrm{FEM}}^{1}$ (the largest vertical displacement of FEM elements of sensing probe from the nondeformed position), the total deformation $O_{\mathrm{FEM}}^{2}$ (displacement summation of FEM elements of sensing probe from the nondeformed position), and the deformation area $O_{\mathrm{FEM}}^{3}$ (the projected area of the deformed surface of the sensing probe), as shown in Figure 11C. The tactile data are necessary to relate FEM tactile data $\left(O_{\mathrm{FEM}}^{1},O_{\mathrm{FEM}}^{2},O_{\mathrm{FEM}}^{3}\right)$ and TSIS tactile data $\left(O_{\mathrm{TSIS}}^{1},O_{\mathrm{TSIS}}^{2},O_{\mathrm{TSIS}}^{3}\right).$ The definitions of TSIS tactile data are as follows: The maximum pixel value $O_{\mathrm{TSIS}}^{1}$ is defined as the pixel value in the centroid of the tactile data. The total pixel value $O_{\mathrm{TSIS}}^{2}$ is defined as the summation of pixel values in the tactile data. The deformation area of pixel $O_{\mathrm{TSIS}}^{3}$ is defined as the number of pixels greater than the specific threshold value in the tactile data. The inversion algorithm was used to estimate $\left(\hat{d},\hat{h},\hat{E}\right)$ using the determined $\left(O_{\mathrm{TSIS}}^{1},O_{\mathrm{TSIS}}^{2},O_{\mathrm{TSIS}}^{3}\right)$. In this method, the multilayered ANN was considered as an inversion algorithm, as shown in Figure 11D.

Despite these methods, algorithms design of tactile perception in the minimally invasive surgery is still a new subject, with related little research, although we hold the opinion that this field could be considerably promising due to the application need.

## Conclusion

Minimally invasive surgery has been the preferred surgery approach owing to its advantages over conventional open surgery. Tactile information has been proven effective to improve surgeons’ performance, while most reviews for MIS were only focusing on force sensors and force feedback, neglecting other tactile information. In this paper, we reported tactile sensors, tactile perception algorithms, and tactile perception applications for MIS. These include a description of various tactile sensors and feedbacks not limited to force sensors and force feedback, the state-of-the-art and novel machine learning algorithms in tactile images for tactile perception in MIS, and potential tactile perception applications for MIS, especially for detecting tissue properties. Finally, this review contains some of the limitations and challenges of each technical aspect.

An emerging research and development trend in the literature is the fusion of various tactile information. Utilizing force information alone has met challenges, including low effectiveness in the algorithm level, the attenuation of the force signal, and amplified disturbing force. Therefore, some studies aimed to develop hybrid sensors employing more than merely one sensing principle to measure one or multiple physical stimuli (e.g., force, slippage, stiffness, etc.) to obtain more robust measurements of physical stimuli and cover wider working environments. With the development of tactile sensors of various sensing modalities, some novel tactile feedback systems were reported (e.g., graphical display system, audio display system, etc.). Some researchers attempted to obtain more tactile information at the algorithm level. Inspired by computer vision technologies, some researchers reported machine learning algorithms for obtaining more than merely one kind of tactile information from tactile images, where a tactile element is treated as an image pixel. Tactile perception algorithms design in MIS is still a new subject, with related little research; while considering the high accuracy, high robustness, and excellent real-time performance of machine learning algorithms, we hold the opinion that this field could be considerably promising due to the application need.

## Author Contributions

All authors researched the literature, drafted, and wrote the review article, and approved the submitted version.

### Conflict of Interest

The authors declare that the research was conducted in the absence of any commercial or financial relationships that could be construed as a potential conflict of interest.
